# An exceptionally preserved Eocene shark and the rise of modern predator–prey interactions in the coral reef food web

**DOI:** 10.1186/s40851-016-0045-4

**Published:** 2016-04-01

**Authors:** Federico Fanti, Daniela Minelli, Gabriele Larocca Conte, Tetsuto Miyashita

**Affiliations:** Dipartimento di Scienze Biologiche, Geologiche e Ambientali, Alma Mater Studiorum, Università di Bologna, Via Zamboni 67, Bologna, 40126 Italy; Museo Geologico Giovanni Capellini, Alma Mater Studiorum, Università di Bologna, Via Zamboni 63, Bologna, 40126 Italy; Department of Biological Sciences, University of Alberta, Edmonton, Alberta T6G 2E9 Canada

**Keywords:** Early eocene climatic optimum, Carcharhinuformes, Triakidae, *Galeorhinus*, *Sphyraena*, Bolca, Soft tissue, Stomach content, Nursery habitat, von Bertalanffy

## Abstract

**Background:**

Following extreme climatic warming events, Eocene Lagerstätten document aquatic and terrestrial vertebrate faunas surprisingly similar to modern counterparts. This transition in marine systems is best documented in the earliest teleost-dominated coral reef assemblage of Pesciara di Bolca, northern Italy, from near the end of the Eocene Climatic Optimum. Its rich fauna shows similarities with that of the modern Great Barrier Reef in niche exploitation by and morphological disparity among teleost primary consumers. However, such paleoecological understanding has not transcended trophic levels above primary consumers, particularly in carcharhiniform sharks.

**Results:**

We report an exceptionally preserved fossil school shark (*Galeorhinus cuvieri*) from Pesciara di Bolca. In addition to the spectacular preservation of soft tissues, including brain, muscles, and claspers, this male juvenile shark has stomach contents clearly identifiable as a sphyraenid acanthomorph (barracuda). This association provides evidence that a predator–prey relationship between *Galeorhinus* and *Sphyraena* in the modern coral reefs has roots in the Eocene. A growth curve of the living species of *Galeorhinus* fitted to *G. cuvieri* suggests that all specimens of *G. cuvieri* from the lagoonal deposits of Bolca represent sexually and somatically immature juveniles.

**Conclusion:**

The modern trophic association between higher-degree consumers (*Galeorhinus* and *Sphyraena*) has a counterpart in the Eocene Bolca, just as Bolca and the Great Barrier Reef show parallels among teleost primary consumers. Given the age of Bolca, trophic networks among consumers observed in modern coral reefs arose by the exit from the Climatic Optimum. The biased representation of juveniles suggests that the Bolca Lagerstätte served as a nursery habitat for *G. cuvieri*. Ultraviolet photography may be useful in probing for exceptional soft tissue preservation before common acid preparation methods.

**Electronic supplementary material:**

The online version of this article (doi:10.1186/s40851-016-0045-4) contains supplementary material, which is available to authorized users.

## Introduction

Fossil record documents profound changes in both marine and terrestrial ecosystems across the Paleocene-Eocene Thermal Maxima (55.8 and 53.7 million years ago) [1–4]. These changes are preludes to the modern vertebrate faunas, with the increasing dominance of acanthomorph teleosts in marine systems and the emergence of artiodactyls, perissodactyls, and primates in terrestrial systems, among other radiation and extinction events [1, 3]. It still remains an open question as to whether, how, and exactly what modern ecological traits and feeding interactions arose in such biodiversity hotspots as coral reefs during this time. These fundamental questions demand case studies of ecological traits in the fossil counterparts against those in components of living coral reef vertebrate fauna, but direct evidence that allows such one-to-one comparison is rare.

Pesciara di Bolca Konservat–Lagerstätte from northern Italy occupies a special place in understanding the shift toward modern marine ecosystems. This late Ypresian Lagerstätte with more than 250 vertebrate species (approximately 90 families) [5–7] coincides chronologically with the latest phase of the Early Eocene Climatic Optimum [2, 8, 9] and that of the ‘reef gap’ in which foraminiferal/algal banks and shoals replaced coral-dominated reefs globally across low latitudes (57–42 mya) [2, 10–12]. The locality also sat in the Tethys Sea connecting the Atlantic and Indo–Pacific oceans [13, 14]. Documenting the earliest occurrences of many acanthomorph lineages, the Bolca fishes represent the earliest of clearly defined coral reef fish assemblages [6, 15–17]. This assemblage resembles modern coral reef faunas in its remarkably high functional diversity of primary consumers, which in large part was facilitated by significant increase in morphological disparities among acanthomorph teleosts during the Paleocene-Eocene interval [3, 18–21].

Despite the wealth of ecomorphological data on primary consumers, chondrichthyans from this latest phase of the Climatic Optimum have received little attention. Most chondrichthyans are secondary or tertiary consumers or apex predators, and those from the Bolca Lagerstätte should be no exception [5, 22]. However, the overall morphology of chondrichthyans cannot be readily compared with that of teleosts using same anatomical landmarks in a single ecomorphological analysis, and direct evidence of trophic interactions has been lacking. Consequently, the teleost-centered paleoecological understanding of the Early Eocene vertebrate fauna has not readily transcended trophic levels above primary consumers. Several factors present a challenge to incorporating sharks into the paleoecological scheme, including documentation of head and body morphology, test of sexual dimorphism, and inference of trophic relationships. We solve these problems with the finely preserved specimens of the extinct carcharhiniform shark *Galeorhinus cuvieri* from Pesciara di Bolca Konservat-Lagerstätte. One of the specimens (MGGC 1976) receives a special focus for its exquisite preservation of soft tissues and for its stomach content.

### List of institutional abbreviations

**BM**, Museo dei Fossili di Bolca, Bolca (Verona), Italy; **MCSNV**, Museo Civico di Storia Naturale, Verona, Italy; **MGGC**, Museo Geologico Giovanni Capellini, Bologna, Italy; **MGP-PD**, Museo di Geologia e Paleontologia, Padova, Italy.

## Methods

The restoration project for damage caused by a 2012 earthquake allowed close examination of MGGC 1976 (slab and counterslab) using natural color and ultraviolet photography, stereomicroscopy, X-ray computed tomography, and SEM of extracted samples. The systematic use of UV light distinguished preserved tissues from cryptic reconstruction with fine grout and organic pigments in the 18th century. Furthermore, black light exposure revealed tissue–specific fluorescence, which delineated individual cartilages, muscles, brain, and visceral structures. We tested these interpretations under natural light, using differences in texture, topographical features, and colors.

**Fig. 1:**
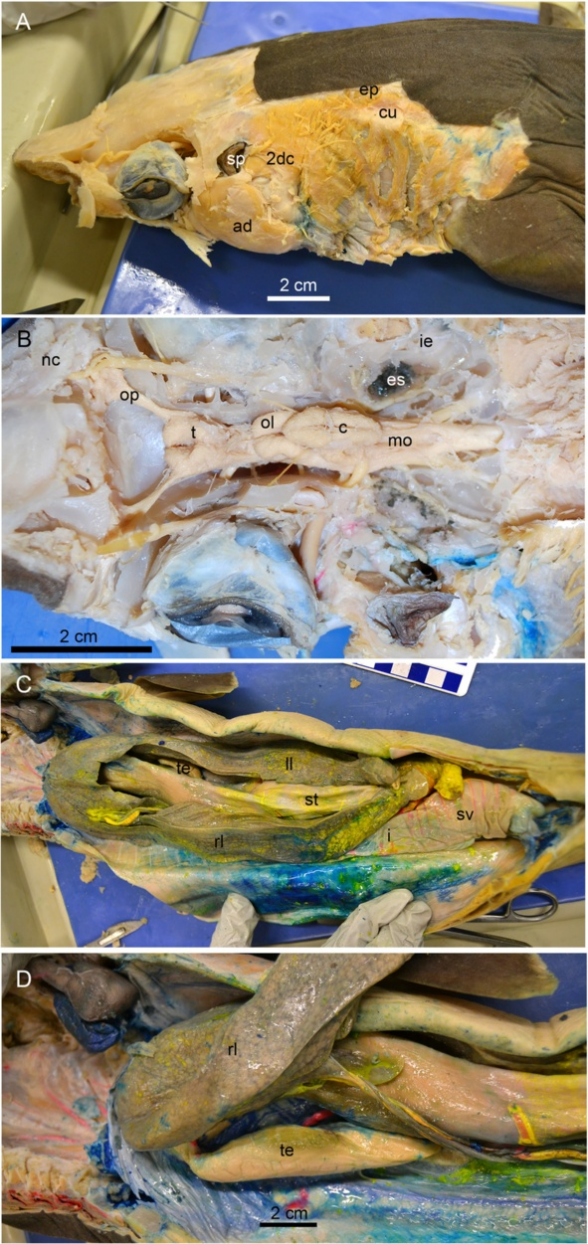
A dissected male spiny dogfish (*Squalus acanthias*), showing the soft tissues identified in MGGC 1976 (*G. cuvieri*). **a** The head in left dorsolateral view with the skin removed. **b** The endocranial cavity in dorsal view with the chondrocranial roof removed. **c** The abdominal region in ventral view. **d** The abdominal region in right ventrolateral view. Abbreviations: *2 dc*, second dorsal constrictors; *ad*, adductor mandibulae; *cu*, cucullaris; **d**, diencephalon; *ep*, epaxial muscles; *es*, endolymphatic sac; *i*, intestine; *ie*, inner ear; *ll*, left liver; *mo*, medulla oblongata; *nc*, nasal cavity; *ol*, optic lobe; *op*, olfactory peduncle; *rl*, right liver; *sp*, spiracle; *st*, stomach; *sv*, spiral valve; *t*, telencephalon; *te*, testis

We set three criteria for identification of the preserved elements: (1) clearly delineated edges under both visible and ultraviolet light, which are not obscured by more than 50 % of the outline; (2) area distinguished by texture, topography, and/or color enclosed within the outline; and (3) topographical and morphological consistency with corresponding elements in the living sharks (Fig. 1). As *G. galeus* is a FAO-listed vulnerable species, we used males of the living carcharhiniforms *Squalus acanthias* and *Mustelus mustelus* for dissection.

Ultraviolet photography could improve screening for soft tissue preservation in specimens from Lagerstätten like Pesciara di Bolca. Although it is a common practice to prepare such specimens using acid solution [23], dissolution of matrix can lead to loss of preserved soft tissues or impressions.

## Results

### Systematic paleontology

Carcharhiniformes Compagno, 1973

Triakidae Gray, 1851

*Galeorhinus* Blainville, 1816

#### Emended diagnosis

A carcharhiniform selachian with a unique combination of characters assembled from descriptions of living and fossil species [24, 25]: mouth arcuate, with sides of lower jaw convex; pectoral fin greater in area than first dorsal fin; distance from snout tip to first dorsal origin much greater than interdorsal space; supraorbital crest moderately concave; teeth up to 5 mm tall; cusp bent toward the rear (from anterior files distally); upper anterolateral teeth low–crowned; mesial cutting edge much greater than distal cutting edge; distinct cusplets of decreasing size on distal heel, well–separated from cusp; thin root with a distinct furrow; basal edge weakly concave.

*Galeorhinus cuvieri* (Agassiz, 1835)

**Fig. 2:**
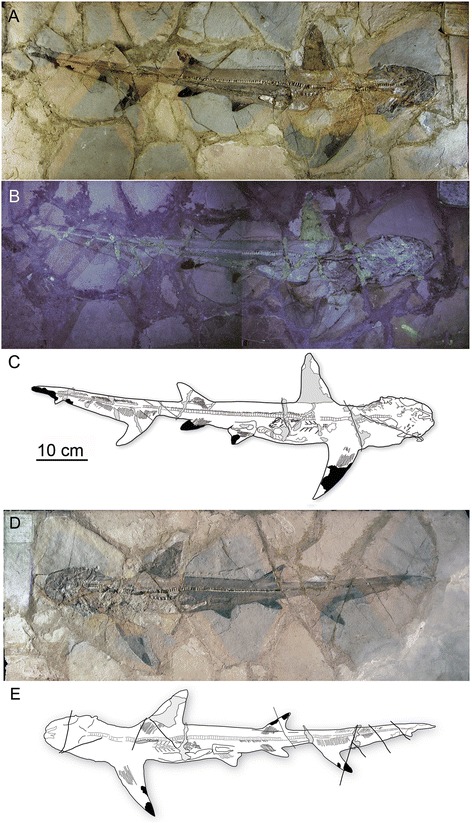
MGGC 1976, a juvenile male of *Galeorhinus cuvieri* in dorsal to right lateral view. Photographs of the main slab under natural (**a**) and UV light (**b**) and an interpretive drawing of anatomical structures (**c**) with artifacts in light grey and areas preserved with dermal denticles in black. Photographs of the counterslab under natural light (**d**) and an interpretive drawing of anatomical structures (**e**) with artifacts in light grey and areas preserved with dermal denticles in black

**Fig. 3:**
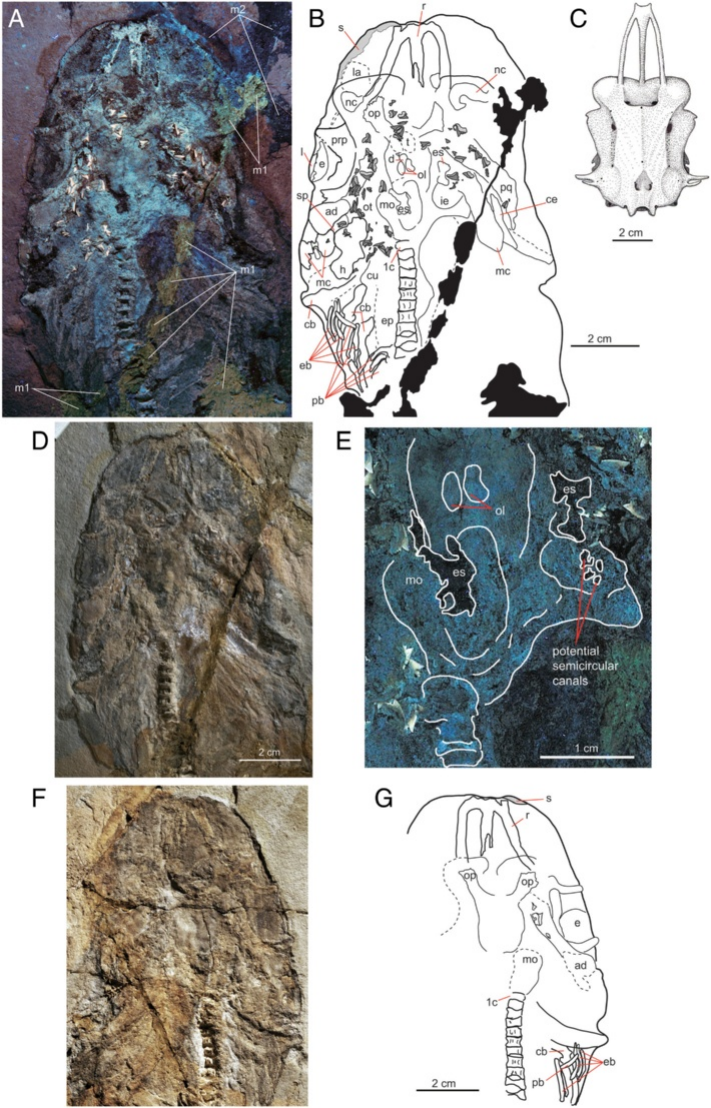
Head anatomy of MGGC 1976 (*G. cuvieri*) with exceptional preservation of soft tissues. Photograph of the head region of the main slab under UV light (**a**) and interpretive drawing of anatomical structures (**b**) in dorsal view, compared to the chondrocranium of the living *G. galeus* (**c**) in dorsal view. The chondrocranium (**c**) is modified from [25] and scaled to the same anteroposterior length. The slab was assembled from multiple parts using mortar glue (*m1*) and reconstructed using pigmented mortar (*m2*). **d** Photograph of the head region of the main slab under natural light. **e** Details of the endocranial cavity and the braincase under UV light. **f, g** The head region of the counter slab under natural light (**f**) and outline (**g**). Abbreviations: *1c*, first cervical vertebra; *ad*, adductor mandibulae; **c**, cerebellum; *cb*, ceratobranchial; *ce*, ceratohyal; *cu*, cucullaris; **e**, eye; *eb*, epibranchials; *ep*, epaxial muscle; *es*, endolymphatic sac; **h**, hyomandibular overlapped by second dorsal constrictors; *ie*, inner ear; **l**, lens; *la*, sensory area with the ampullae of Lorenzini; *m1*, mortar used to glue parts of the slab; *m2*, pigmented mortar used to reconstruct anatomical parts; *mc*, Meckel’s cartilage; *mo*, medulla oblongata; *nc*, nasal cavity; *ol*, optic lobes; *op*, olfactory peduncle; *ot*, otic capsule; *pb*, pharingobranchial; *pq*, palatoquadrate; *prp*, preorbital process; *r*, rostrum; *s*, patches of dermal denticles; *sp*, spiracle

**Fig. 4:**
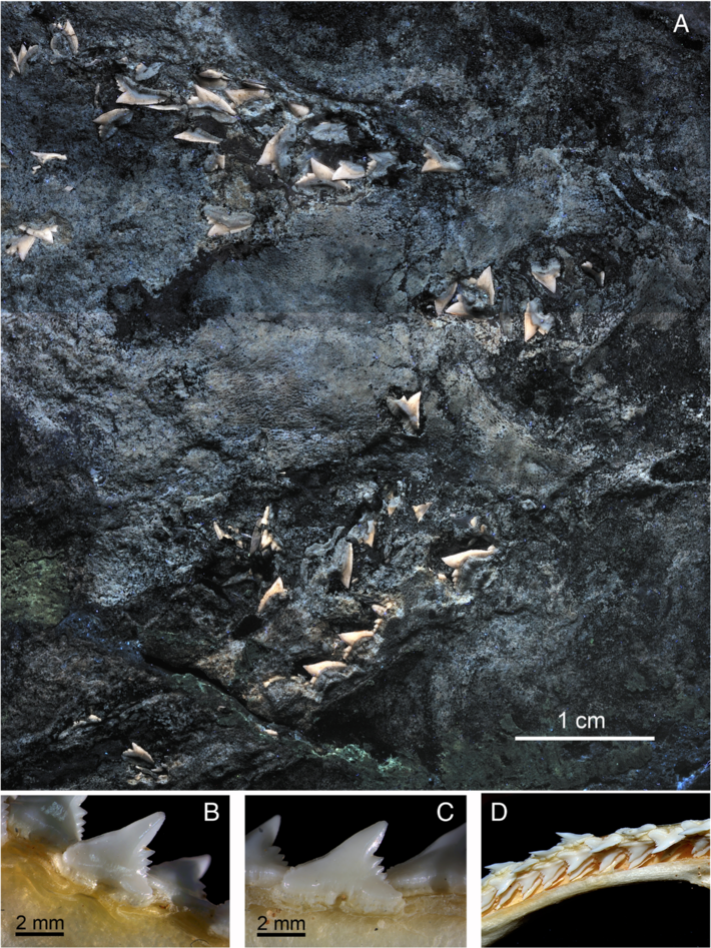
Tooth morphology of the extinct and living species of *Galeorhinus*, *G. cuvieri* MGGC 1976 (**a**) and *G. galeus* (**b**-**d**). **a** A composite image of UV photographs showing the upper and lower tooth arcades of MGGC 1976. **b**-**d** Detailed photographs of the dentition of *G. galeus*, showing **b** a right upper anterior tooth (tooth position: #3), **c** right upper anterolateral tooth (#5), and left upper anterolateral to lateral tooth files (**d**). Arrow indicates some of the characters differentially diagnostic to *G. cuvieri* (see Diagnosis). Ch. 1: tooth cusp upturned near tip (from anterior files distally); Ch. 2: distal and mesial shoulders of tooth at similar horizontal levels; Ch. 3: distal heel separated from cusp by a notch deeper than cusplets measured along distal cutting edge; Ch. 4, relatively wide basal furrow

**Fig. 5:**
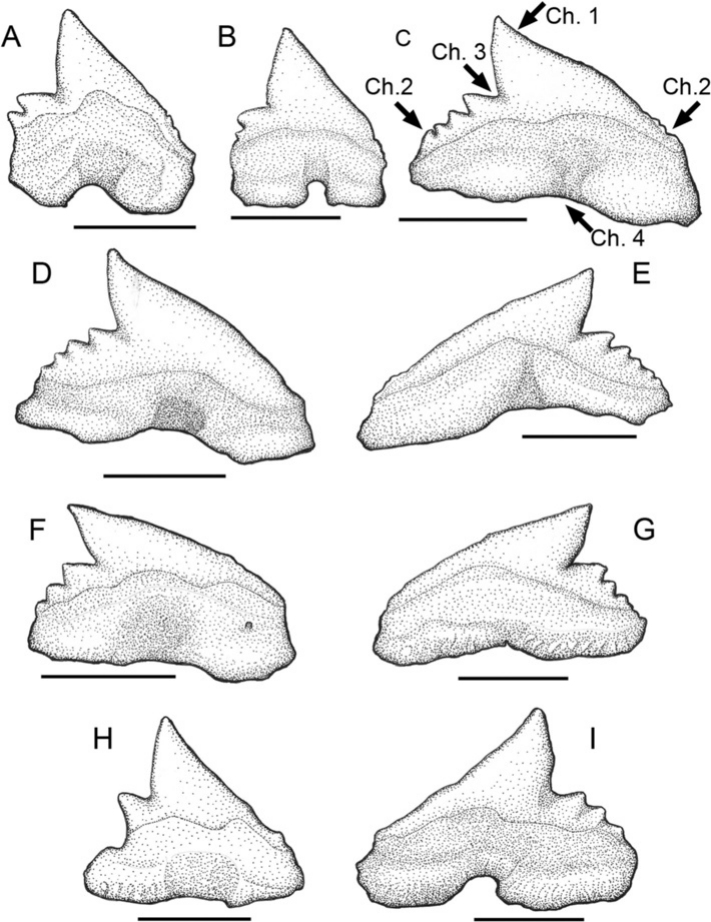
Representative teeth of MGGC 1976 (*G. cuvieri*). Interpretive drawings of two left lower anterior teeth (**a**, **b**), two left lower anterolateral teeth (**c**, **d**), three left lower lateral teeth (**e**-**g**), a right upper anterior tooth (**h**) and a left upper anterior tooth (**i**). MGGC 1976 incompletely preserves both right and left pairs of upper and lower tooth series. Scale bar = 2 cm

**Fig. 6:**
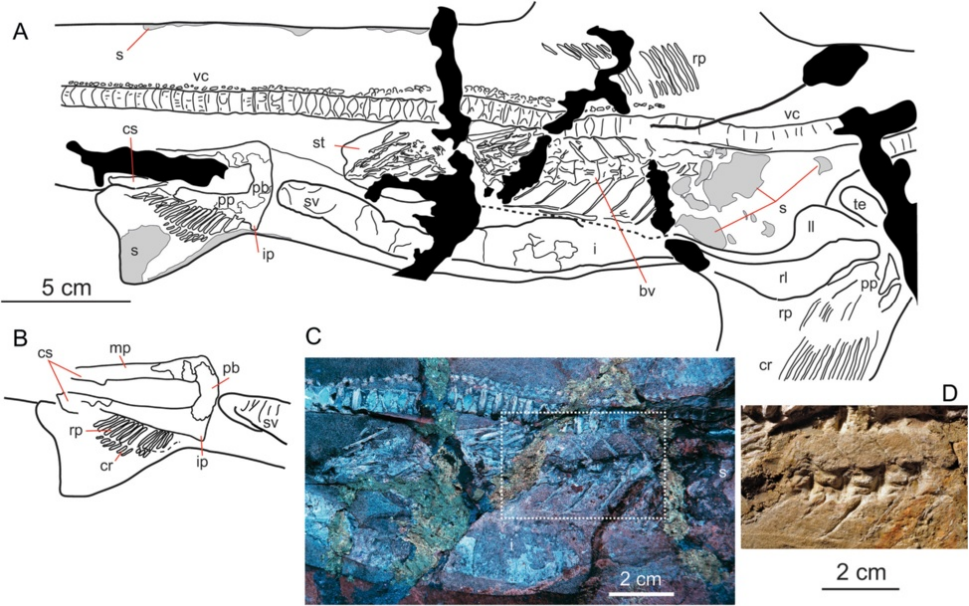
Visceral anatomy and stomach content of MGGC 1976 (*G. cuvieri*) with exceptional preservation of soft tissues. **a** An interpretive drawing of the abdominal region of MGGC 1976 on the main slab, showing the visceral tissues, pelvic girdle, and stomach content. **b** An interpretive drawing of the pelvic girdle of MGGC 1976 on the counterslab. **c** Photograph under UV light of the stomach and intestine, revealing an undigested partial skeleton of the barracuda *Sphyraena bolcensis*. **d** Photograph under natural light to show the detailed morphology of the caudal vertebrae of *S. bolcensis*. Abbreviations: *bv*, caudal vertebrae of *S. bolcensis*; *cr*, ceratotrichia; *cs*, claspers; *i*, intestine; *ip*, iliac process; *ll*, left liver; *mp*, metapterygium; *pb*, puboischiatic bar; *pp*, propterygium; *rl*, right liver; *rp*, radial pterigyophores; *s*, dermal denticle patches; *st*, stomach; *sv*, spiral valve; *te*, testis; *vc*, vertebral column

**Fig. 7:**
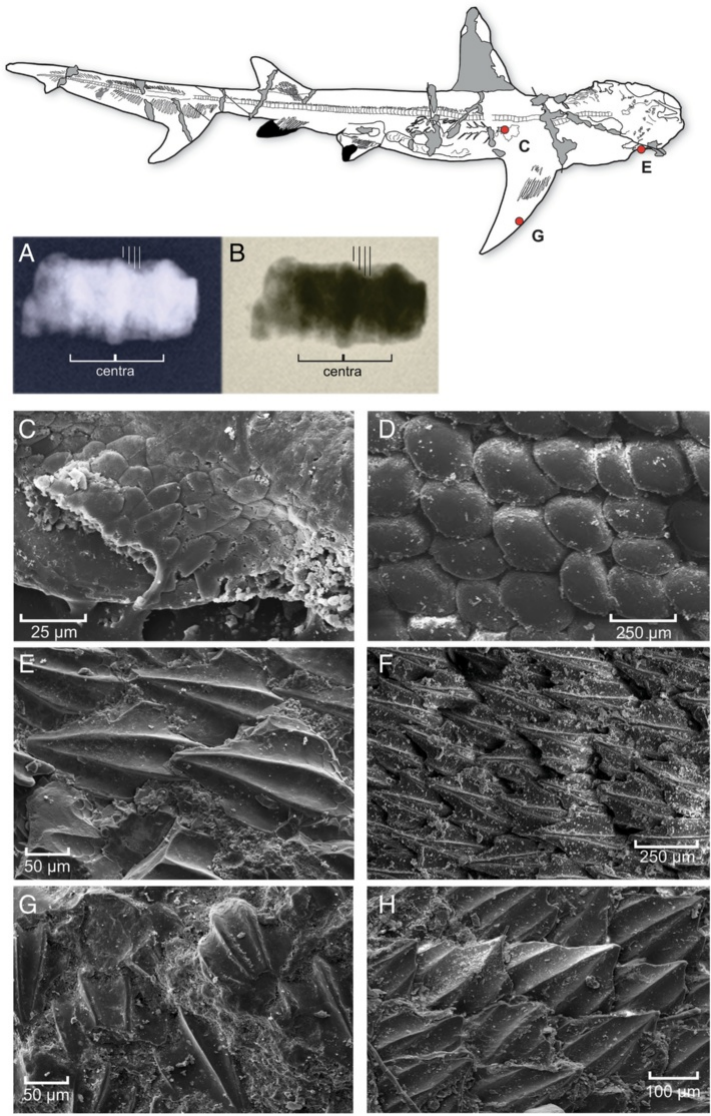
Additional information on the morphology of *G. cuvieri*, with emphasis on dermal denticles in the extinct and living species of *Galeorhinus*. **a, b**, X-ray radiograph of two vertebral centra sampled from a young female of *G. cuvieri* (MCSNV T 1124), showing four vertebral bands. *G. cuvieri* MGGC 1976 (**c**, **e**, **g**) and *G. galeus* (**d**, **f**, **h**). An interpretive drawing of MGGC 1976 shows areas of preserved dermal denticles sampled for scanning electron microscopy (SEM). **c**, **d** SEM micrographs of dermal denticles from the abdominal region in *G. cuvieri* (**c**) and *G. galeus* (**d**). **e**, **f** SEM micrographs of dermal denticles from the head region in *G. cuvieri* (**e**) and *G. galeus* (**f**). **g**, **h** SEM micrographs of dermal denticles from the pectoral fin in *G. cuvieri* (**g**) and *G. galeus* (**h**)

**Fig. 8:**
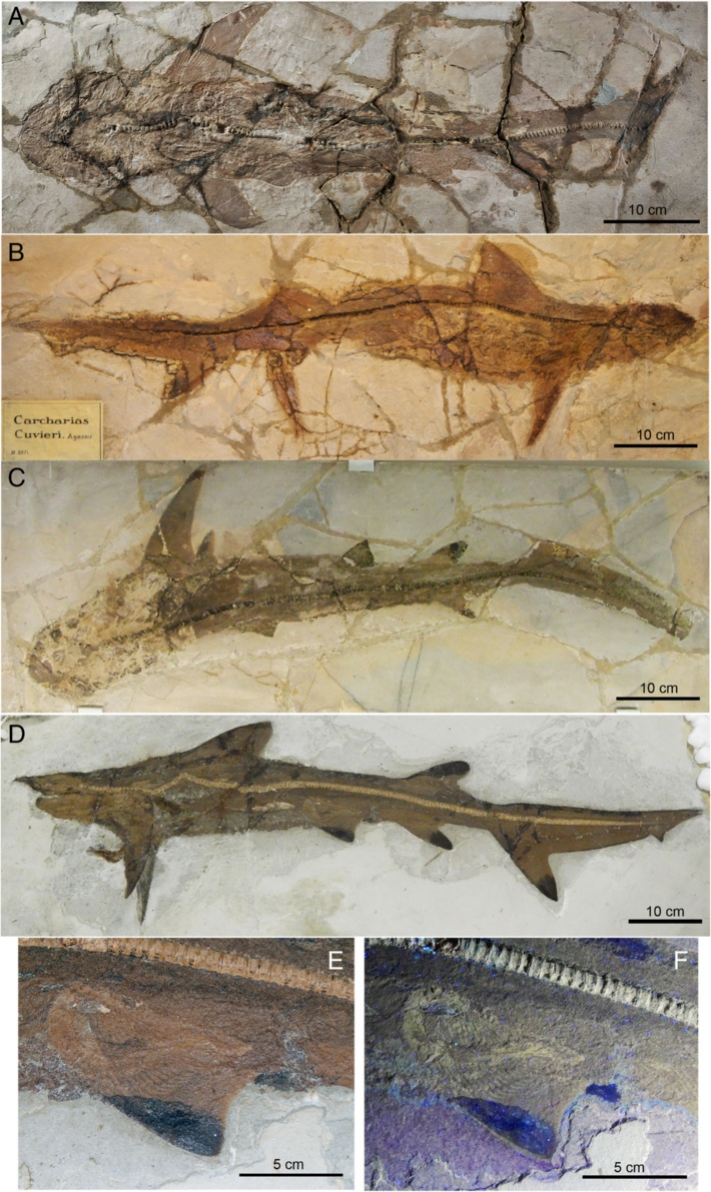
Specimens of *G. cuvieri* with whole body preservation from Pesciara di Bolca. **a**, MNHN F Bol516 (holotype, sex undetermined; photograph provided by G. Clément). **b**, MGP-PD 8871C (sex undetermined). This specimen is the smallest among those we examined and preserved with digested and unidentifiable stomach contents. **c**, MCSNV VII B96 (sex undetermined). **d**-**f**, MCSNV T.1124 (juvenile female) in left lateral view (**d**) and detailed view of the pelvic girdle under natural (**e**) and UV (**f**) light. [planned for single column width: 3.54 inches]

Figs. 2, 3, 4, 5, 6, 7 and 8

#### Referred specimens

MNHN F.Bol.516 (holotype) (Fig. 8a), MCSNV T 1124 (Fig. 8d–f), MCSNV VII B 96-97 (Fig. 8c), MGGC 1976 (Figs. 2, 3, 4, 5, 6 and 7), MGP-PD 8871C/8872C (Fig. 8b), BM B70.

#### Locality and horizon

Pesciara di Bolca, Veneto, Italy (upper Ypresian; SBZ 11; NP 14).

#### Emended diagnosis

**Fig. 9:**
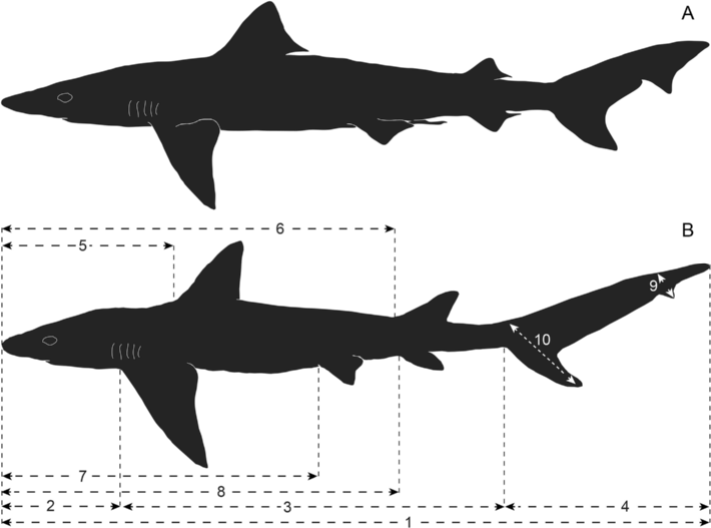
Comparison of body proportions and outlines between the living and extinct species of *Galeorhinus* in left lateral view. **a** Mature male of the living *G. galeus* (modified from [25, 41]). **b** Juvenile male of the extinct *G. cuvieri* reconstructed on the basis of MGGC 1976, showing standard metric traits for Additional file 1: Table S1. Relative to *G. galeus*, *G. cuvieri* has a shorter rostrum, larger pectoral fins, taller second dorsal fin, shorter interdorsal distance, longer caudal fin, smaller accessory lobe, and smaller ventral lobe of caudal fin. Not to scale

*Galeorhinus* with the following unique combination of features: relatively short rostrum (not significantly longer than orbital length) (Figs. 2 and 9); tooth cusp upturned near tip (from anterior files distally) (Fig. 5; Character [Ch.] 1); distal and mesial shoulders of tooth at similar horizontal levels (Fig. 5; Ch. 2); distal heel separated from cusp by a notch deeper than cusplets measured along distal cutting edge (Fig. 5; Ch. 3); relatively wide basal furrow (Fig. 5; Ch. 4); vertebral count greater than 200 (Fig. 2); second dorsal fin taller than half the height of first dorsal (Figs. 2 and 9); dorsal edge of caudal fin as long as or longer than interdorsal distance (Figs. 2 and 9); accessory lobe of caudal fin less than a quarter of area of distal rest of main lobe (Figs. 2 and 9).

### Description

#### Overall morphology

MGGC 1976 (the best-preserved specimen of *G. cuvieri*) lies on the right side of the body (main slab: upper; counterslab: lower), with the head twisted clockwise (Fig. 2). The pectoral fin has approximately twice the area of the first dorsal fin, and this ratio is greater than in the living species of *Galeorhinus* [25]. The caudal fin occupies a third of the entire body length, and its dorsal lobe has an accessory lobe. In comparison to the living species of *Galeorhinus*, the caudal fin is slender and the accessory lobe is small. The pelvic girdle extends beyond the pelvic fin posteriorly into a clasper, which indicates that MGGC 1976 is a male (Fig. 6b).

MGGC 1976 (92 cm in total length) has a vertebral count of between 208 and 213 (114 to 118 in the trunk, 94–95 in the tail region). In comparison, MCSNV T 1124 (Fig. 8d–f) is a female of the same species (pelvic girdle clearly lacking claspers) and has a vertebral count between 200 and 204 (104 to 106 in the trunk, 96 to 98 in the tail). The vertebral count exceeding 200 in *G. cuvieri* is significantly higher than that in the living galeorhines (*G. galeus*: 136 centra, 83 in the trunk, 53 in the tail; *G. japonicus*: 160 centra, 106 in the trunk, 54 in the tail). Across the living selachians, vertebral counts of greater than 200 are rare. The exceptions are several carcharinid genera (*Carcharhinus*, *Galeocerdo*, *Prionace*, *Triaenodon*) and the leptochariid *Leptocharias* [25]. Dermal denticles are preserved in patches across the body (Fig. 7).

#### Chondrocranium and branchial skeleton

The tripartite rostrum of MGGC 1976 is relatively short among triakid sharks, not significantly longer than the orbital length (Fig. 3). The neurocranium is preserved with a clear outline on the left side of the head. The sigmoidal nasal cavity is entirely exposed on the right side and the sensory area for the ampullae of Lorenzini on the left side. Both right and left Meckel’s cartilages can be delineated along the tooth series, meeting in roughly 70° (Fig. 3). The ceratohyals sit close to the proximal ends of the lower jaws. The palatoquadrates are preserved nearly in parallel to the Meckel’s cartilages. On the left side of the head, a spiracle sits between the palatoquadrate and the hyomandibula. A full set of pharyngobranchials and epibranchials overlaps with at least three exposed ceratobranchials.

#### Dentition

The teeth of MGGC 1976 are small (3–4 mm crown height), cuspidate, and not bladelike (Figs. 4 and 5). Unlike the coeval *Eogaleus* [26], the crown height is never greater than the fore-aft basal length across the tooth series, even near the symphysis. From anterior files distally, the teeth have a cusp bent more posteriorly than dorsally so that the mesial cutting edge is greater than the distal edge. The cusp is upturned near the tip, which differs from other extinct species of *Galeorhinus* such as *G. gomphorhiza*, *G. minutissimus*, and *G. ypresiensis* and from other triakid genera such as *Khouribgaleus*, *Palaeogaleus*, and *Triakis* [24, 25]. A similarly upturned outline is also present in *G. duchaussoisi*, *G. goncalvesi*, *G. louisi*, and *Iago angustidens* [24, 27, 28]. However, the teeth of *G. cuvieri* have dorsoventrally taller roots than the teeth of *G. goncalvesi*; have smaller cusplets relative to the cusp than in *G. duchaussoisi* and *G. louisi*; and are overall lower apicobasally than those of *I. angustidens*. Unlike *G. duchaussoisi* and *Hypogaleus* [25, 28], the mesial and distal shoulders are at similar horizontal levels across the tooth series. From the anterior files distally, the distal heel develops distinct cusplets of decreasing sizes. The number and size of the cusplets vary from tooth to tooth (one in symphyseal to five in lateral; Figs. 4 and 5), but they are invariably oriented posterodorsally, irrespective of the orientation of the cusp. There are no more than five distal cusplets. The count of distal cusplets in *G cuvieri* is greater than in *G. mesetaensis* and *G. minutissimus* (less than three), but smaller than in *G. duchaussoisi* (up to six). The distal series of cusplets is separated from the cusp by a notch deeper than the distal cutting edge of the first cusplet — a unique feature among triakids with the exceptions of *Hypogaleus* and *Khouribgaleus* [25, 29]. *G. duchaussoisi* has a similarly deep notch in some of the anterior teeth, but these cusplets are larger relative to the cusp in this taxon than in *G. cuvieri* [28]. The teeth of *G. duchaussoisi* are also more asymmetric mesiodistally than those of *G. cuvieri*. In the anterior to anterolateral teeth of *G. cuvieri*, the mesial cutting edge has weakly developed denticles, but none of them is a distinct cusplet. This feature distinguishes *G. cuvieri* from other species of *Galeorhinus* such as *G. ypressiensis* [28]. The basal edge is weakly concave, and the basal furrow is as wide as the first distal cusplet.

These features distinguish the dentition of *G. cuvieri* from that of other triakids [24–31]. *G. cuvieri* differs from all other species of *Galeorhinus* in having the deep notch separating the cusp and the first distal cusplet, among other features that differentially sets *G. cuvieri* apart from each of the species of *Galeorhinus* in various combinations. The cusp is taller than at least three times the height of the first cusplet in *G. cuvieri* unlike *Hemitriakis*, *Hypogaleus*, *Mustelus*, *Pachygaleus*, and *Triakis*. The presence of multiple distinct distal cusplets in all but symphyseal teeth rules out *Furgaleus*, *Iago*, *Paratriakis*, *Scylliogaleus*, and *Triakis*, whereas the absence of parallel apicobasal ridges precludes *Archaeotriakis*, *Mustelus*, *Palaeogaleus*, *Squatigaleus*, *Triakis*, and *Xystrogaleus*.

#### Nervous system

Centered in the head of MGGC 1976, an area of intense UV reflection in contrast to the less reflective surrounding matrix indicates that the preserved structure is not simply an infilling of the endocranial cavity. Instead, it likely represents permineralization in the cerebrospinal space between the meninx and brain (M. I. Coates, pers. commn. 2015). Remarkable preservation allows identification of major external features in the central and peripheral nervous systems (Fig. 3), which can be delineated with both highly reflective areas under UV light and texture/topography under natural light. The olfactory tracts (CN I) extend anteriorly in the approximate angle of 90° from each other. The right cerebral hemisphere sits above the base of the olfactory tract, although the left counterpart is obscured by the underlying teeth. These parts of the telencephalon are followed posteriorly by a pair of the optic lobes, and finally the cerebellum that sits on the medulla oblongata.

Within the left orbit, the eye and lens are clearly demarcated both in texture and outline.

Just anterior to the left preorbital process, an area with strongly UV-reflective dots may represent the chemosensory field of the ampullae of Lorenzini. On the right side of the head, the otic capsule is preserved with possible traces of semicircular canals. These potential semicircular canals are distinguished as narrow UV-nonreflective loops within an intensely UV-reflective area. They appear to represent the proximal third of the anterior, posterior, and horizontal loops. Anterior to the otic capsule, an area filled with the non-UV reflective matrix represents the endolymphatic sac. Its potential counterpart on the left side sits on top of the medulla oblongata and cerebellum, presumably shifted as the chondrocranium was crushed.

#### Musculature

In MGGC 1976, several major muscles are visible both under natural and UV light on the left side of the head and in the branchial region (Fig. 3). The UV-reflective structure overlapping the left palatoquadrate and Meckel’s cartilage represents the adductor mandibulae, a major jaw adductor that attaches to these two cartilages and occupies a large area of the head behind the postorbital process in lateral view. A similarly UV-reflective structure overlapping the hyomandibula posterior to the spiracle represents a second dorsal constrictor (levators hyomandibulae and hyoideus). Near the left series of the gill arches, two unclearly defined structures extend in parallel with the vertebral column. If these are muscles, the lateral element would represent the cucullaris, whereas the medial one would be the epaxial musculature.

#### Visceral tissues

The abdomen of MGGC 1976 is preserved in right lateral view on the main slab (Fig. 6). The stomach overlaps the large left liver, whereas the smaller right liver partly parallels the ventral margin of the stomach. The testis sits near the proximal end of the livers. The intestine follows the stomach in the sigmoidal twist on the left side of the body. Once at the midline, it parallels the ventral margin of the stomach posteriorly. The helical contents reveal that it was a spiral valve, as in the living selachians. Count of the spiral valves is six or more, slightly greater than that in the living species of *Galeorhinus* at 4 or 5 [25].

The pelvic girdle of MGGC 1976 has claspers. The stout, slightly anteriorly convex puboischiatic bar bridges the iliac process. The propterygium extends at approximately 80 degrees with respect to the puboischiatic bar. The metapterygium posteriorly extends into a clasper on both sides. This is not the case in MCSNV T 1124, which, therefore, is a female (Fig. 8d-f).

#### Stomach content

The stomach content of MGGC 1976 consists of six articulated distal caudal vertebrae and dismembered caudal fin rays (Fig. 6). The amphicoelous centra are as long anteroposteriorly as tall dorsoventrally (approximately 7 and 8 mm each). In absolute size, the consumed prey had centra as large as those of the consumer (dorsoventral height of 8.2 mm for the centra below the first dorsal fin in MGGC 1976). The neural and haemal spines are semi–equal in length and oriented posterodorsally at approximately 40°. The spine length and the associated body outline show no evidence of tapering toward the caudal fin.

**Fig. 10:**
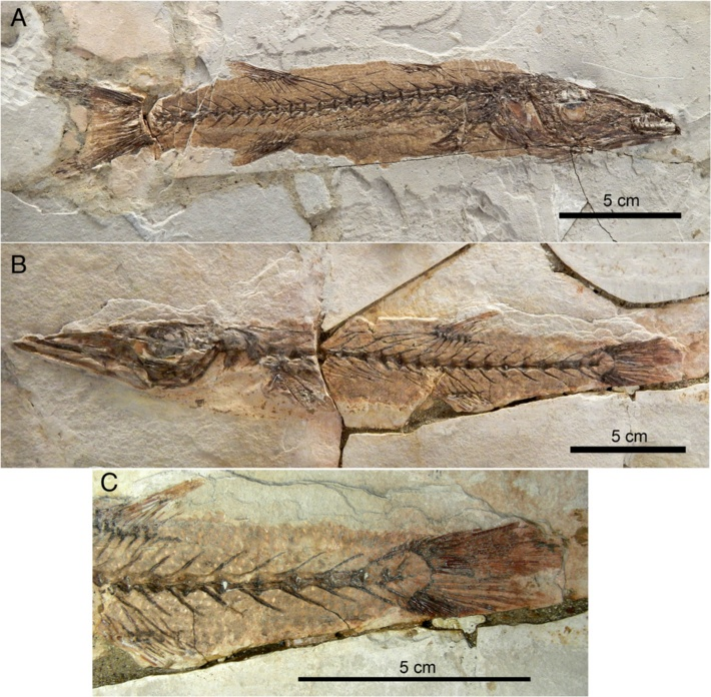
Two specimens of *Sphyraena bolcensis* (Sphyraenidae) from Pesciara di Bolca. This taxon formed part of the diet for *G. cuvieri*. **a** MCSNV VII B18 in right lateral view. **b**, **c** MCSNV VII B12 in left lateral view, with a close up of the caudal region for comparison with the stomach content of MGGC 1976

At this body size, candidates for the consumed fish are limited in the Bolca fauna. We considered scombrids and sphyraenids, both well represented in the locality [5]. The stomach content is not a scombrid, because neural and haemal spines do not decrease significantly in length from anterior to posterior positions along the vertebral column, because the spine orientation is at substantially lower angle around 40°, and because dorsal finlets are absent [32]. The vertebral centra are longer anteroposteriorly than tall dorsoventrally. These morphological features are consistent with the sphyraenid *Sphyraena bolcensis* [33, 34] (Fig. 10). This association does not entirely rule out scavenging, but the intact preservation of an articulated skeleton in the stomach indicates that the preserved portion of *S. bolcensis* was ingested at or near death in a single large bite. Consistent with this, there is no evidence of erosion or weathering on the vertebrae of the prey.

### Age and size estimates

Curiously, all six specimens of *G. cuvieri* fall into a range of similar body sizes from the total length of 54 to 92 cm (Figs. 2 and 8). Perhaps this small variation reflects similar ontogenetic ages among the individuals. We used the von Bertalanffy growth equation [35] to calculate age and mass estimates as routinely applied to modern chondrichthyans [36, 37]:

1 $$ {t}_1=\left(\frac{1}{k}\right) \ln \frac{L_{\infty }}{L_{\infty }-{L}_t}+{t}_0 $$

where *t*_1_ is age of the individual, *k* is a growth coefficient (rate of change of length increment), *L*_*∞*_ is the mean maximum body length for the population, *L*_*t*_ is body length of the individual, and *t*_*0*_ is hypothetical postnatal age extrapolated from a growth curve when length equals zero. We substituted for the parameters *k*, *L*_*∞*_, and *t*_*0*_ with values taken from growth curves of multiple living carcharhiniform populations (Table 1). These calculations provide a range of numerical estimates. For example, a dataset for males of *G. galeus* from Australia [38] has the following life history parameters:

**Table 1:** Age estimates for specimens of *Galeorhinus cuvieri* using the von Bertalanffy functions for living relatives. All of the specimens are predicted to represent juveniles well before — or in some cases near — sexual and somatic maturity

Taxon	S.	*k*	*L*_*∞*_	*t*_0_	Age at maturity	MGGC 1976^M^	MCSNV T.1124^F^	MGP-PD 8871C	MCSNV VII B96	MNHN F Bol516	BM B70	Source
*Galeorhinus galeus*												
Galeorhinus galeus*	M	0.1675	158.33	-1.2545	8+	3.94	3.94	2.16	2.87	2.23	1.24	[38]
Galeorhinus galeus*	F	0.1600	161.83	-1.2818	10+	3.97	3.97	2.19	2.90	2.26	1.26	[38]
Galeorhinus galeus*	C	0.1693	160.04	-1.2699	8-10+	3.95	3.95	2.17	2.88	2.24	1.24	[38]
*Galeorhinus galeus* ^§ R1^	M	0.154	142.9	-1.64	12-17	5.06	5.06	2.64	3.59	2.73	1.44	[39]
*Galeorhinus galeus* ^§ R1^	F	0.086	179.2	-2.68	13-15	5.70	5.70	2.97	4.08	3.08	1.49	[39]
*Galeorhinus galeus* ^§ R1^	C	0.104	165.8	-2.37	12-17	5.41	5.41	2.80	3.85	2.90	1.42	[39]
*Galeorhinus galeus* ^§ R2^	C	0.086	180.4	-2.48	12-17	5.81	5.81	3.13	4.22	3.23	1.66	[39]
*Galeorhinus galeus* ^§ LF^	C	0.131	154.9	-1.91	12-17	4.97	4.97	2.59	3.54	2.68	1.36	[39]
Triakidae												
*Mustelus mustelus*	M	0.12	145.1	-2.14	6-9	6.24	6.24	3.24	4.41	3.35	1.74	[63]
*Mustelus mustelus*	F	0.06	204.96	-3.55	12-15	6.38	6.38	3.29	4.56	3.41	1.55	[63]
*Mustelus mustelus*	C	0.06	198.94	-3.82	6-15	6.53	6.53	3.28	4.61	3.41	1.46	[63]
*Triakis semifasciata*	M	0.089	149.9	-2.31	7+	8.38	8.38	4.62	6.10	4.76	2.71	[64]
*Triakis semifasciata*	F	0.073	160.2	-2.31	10+	9.39	9.39	5.41	7.00	5.56	3.32	[64]
*Triakis semifasciata*	C	0.082	153.6	-2.31	7-10+	8.83	8.83	4.96	6.50	5.11	2.97	[64]
Non-triakid carcharhiniforms												
*Carcharrhinus leucas*	C	0.076	285	-3	M: 14-15	2.13	2.13	0.65	1.27	0.71	-0.24	[65]
					F: 18+							
*Carcharhinus plumbeus*	C	0.046	186	-6.45	30	8.39	8.39	3.63	5.57	3.81	1.01	[66]
*Negaprion brevirostris*	C	0.057	317.65	-2.302	M: 11.6+	3.70	3.70	1.99	2.71	2.07	0.97	[67]
					F: 12.7+							
*Sphyrna lewini*	M	0.131	336.4	-1.09	4.3+	1.35	1.35	0.66	0.95	0.69	0.25	[68]
*Sphyrna lewini*	F	0.156	353.3	-0.633	5.8+	1.30	1.30	0.76	0.99	0.78	0.43	[68]
Non-triakid carcharhiniforms with unrealistic age estimates												
*Galeus sauteri*	M	0.081	70.1	-0.527	9+	—	—	50.76 x	—	80.37 x	17.63	[69]
*Galeus sauteri*	F	0.089	69.8	-0.307	7+	—	—	49.90 x	—	—	16.39	[69]
*Rhizoprionodon taylori*	M	1.337	65.2	-0.41	1	—	—	—	—	—	0.91	[70]
*Rhizoprionodon taylori*	F	1.013	73.2	-0.455	1	—	—	2.37	—	2.63	0.87	[70]
*Scyliorhinus canicula*	M	0.118	87.42	-1.09	6.6+	—	—	12.11	18.74 x	12.58	7.06	[71]
*Scyliorhinus canicula*	F	0.15	75.14	-0.96	7.9+	—	—	15.74	—	16.92	7.49	[71]

The von Bertalanffy growth curves for small non-triakid carcharhiniforms do not fit readily to the specimens of *G. cuvieri*

They either return unrealistic age estimates that are significantly older than the longevity of the living counterpart (x) or are unable to calculate the estimates because the specimens of *G. cuvieri* are larger than the mean maximum length (*L*_*∞*_) of the original population (—). *k* = growth coefficient (rate of change in length increment); *L*_*∞*_ = mean maximum length for the population; *t*_0_ = hypothetical postnatal length (*y* intercept of the growth curve). S. = sex: C = male and female combined; F = female; M = male. Age at maturity is in years

*Australian population; ^§^New Zealand population; ^LF^Length-frequency data; ^R1^Reader 1 (for vertebral bands); ^R2^Reader 2 (for vertebral bands)

$$ k = 0.1675;{L}_{\infty } = 158.33\ \left(\mathrm{cm}\right);{t}_0 = -1.2545\ \left(\mathrm{years}\right) $$

As *L*_*t*_ = 92 (cm) for MGGC 1976, the estimated age of the individual is:

$$ {t}_1 = \left[\frac{1}{0.1675}\times \ln \frac{158.33}{158.33\hbox{-} 92}\right]-1.2545\cong 3.9\ \left(\mathrm{years}\right) $$

With the estimated age, another von Bertalanffy growth equation provides a mass estimate of MGGC 1976:

2 $$ w = {P}_{\infty }{\left[1-{e}^{-k\left({t}_1-{t}_0\right)}\right]}^3 $$

where *P*_*∞*_ is mean maximum weight for the population. Again, we use known values taken from a growth curve based on a dataset for males of *G. galeus* in Australia [38] in this example:

$$ {P}_{\infty } = 44.7\ \left(\mathrm{kg}\right);k = 0.1675 $$

At *t*_*1*_ = 3.940 for MGGC 1976:

$$ w=44.7\times {\left[1-{e}^{-0.1675\left(3.940+1.2545\right)}\right]}^3\cong 8.77\ (kg) $$

We fitted multiple von Bertalanffy growth curves derived from various living carcharhiniforms to six specimens of *G. cuvieri* (Table 1). The data sets for different populations of *G. galeus* suggest the age of 3.9 to 5.8 years for the largest of the specimens (MGGC 1976 and MCSNV T 1124) and that of 2.2 to 3.1 years for the smallest (MGP-PD 8871C). This age estimate roughly agrees with the count of hypermineralized major vertebral bands of MCSNV T 1124 in X-ray radiograph (*n* = 4; Fig. 7a, b).

The biological implication of these estimates lies not in the absolute numerical values, but in the predicted relative age within each of the surrogate populations. When fitted against specimens of *G. cuvieri*, the von Bertalanffy growth curve for the Australian population of *G. galeus* suggests that all specimens of *G. cuvieri* with whole body preservation from Bolca, including MGGC 1976, represent sexually and somatically immature juveniles (Table 1; total body length at sexual maturity in the Australian population of *G. galeus*: males > 120 cm; females > 130 cm [38]). Although *G. galeus* has regional variations in growth rates, the body size at sexual maturity is largely consistent across the species. With the data set for the slow-growing New Zealand population of *G. galeus* [39], the age estimates are numerically different (MGGC 1976: approximately 5.8 years old). However, the specimens of *G. cuvieri* still fell into the range of juveniles (sexual maturity reached after 12 years of age in this population; Table 1). The total body length of 92 cm in MGGC 1976 would indicate age of 7-8 years in the Brazilian population of *G. galeus*, again well before sexual maturity (reached after 12 years of age at body length > 113 cm in males) [40]. Although numerical estimates for *G. cuvieri* vary geographically and from warm to cool waters, the inference of immaturity for all specimens of *G. cuvieri* remains consistent across different data sets for the living *Galeorhinus*.

To our knowledge, this study represents the first application of von Bertalanffy equation to fossil selachians. An important inference borne out in this exercise is probable sexual and somatic immaturity, not numerical values of the estimates. This is because of the assumptions underlying our fitting of the growth curves. These estimates assume that *G. cuvieri* had similar growth parameters to those of *G. galeus*. There is no direct evidence to support this assumption other than the congeneric status. However, the implication for sexually immature status would still be robust to violation of this assumption. When fitting the von Bertalanffy growth curves of other triakids and non-triakid carcharhiniforms, the specimens of *G. cuvieri* represent either sexually immature individuals or individuals near the sexual maturity in those respective taxa (Table 1). The only exceptions are those in which the mean maximum size is substantially smaller than the specimens of *G. cuvieri*. In these small carcharhiniforms, the plateau of the growth curve is well below the sizes of the specimens of *G. cuvieri* so the age estimates are unrealistically high (denoted with ‘x’ or ‘—’ in Table 1).

*Hemitriakis*, *Mustelus*, and *Triakis* may reach sexual maturity in the range of body size exhibited by the specimens of *G. cuvieri* (body length from 54 to 92 cm) among the living triakids (Additional file 1: Table S2). This is intriguing because triakids like *Hemitriakis* and *Hypogaleus* inhabit warmer waters than many of the sampled triakid populations (Table 1) [25]. Unfortunately, there is no robust data set for *Hemitriakis* and *Hypogaleus* to estimate von Bertalanffy growth curve. For this group of relatively small triakids, only the two largest specimens of *G. cuvieri* (MGGC 1976 and MCSNV T.1124) barely overlap the onset of sexual maturity in males of the documented populations of *Mustelus* and *Triakis* (Table 1). MCSNV T.1124 is a female (Fig. 8d-f) so this inference of sexual maturity does not apply to this specimen at the very least. In any case, none of these genera shares morphological characters with *G. cuvieri* that other species of *Galeorhinus* do not, both in the teeth and body proportions [24–26]. It is therefore difficult to consider them as a better analogue to estimate the relative age of *G. cuvieri* than the living *Galeorhinus*.

## Discussion

### Taxonomy and sexual dimorphism

The alpha taxonomy of chondrichthyans from Pesciara di Bolca has a convoluted history. In brief, the six specimens referred to *G. cuvieri* (and to its junior synonyms) were distinguished from the coeval carcharhinid *Eogaleus bolcensis* on the basis of tooth and body morphology [24, 26, 41] despite some attempts for synonymization [42]. Subsequently, many tooth-based extinct species of *Galeorhinus* from other early Cenozoic localities were attributed to the tooth-based extinct carcharhinid *Physogaleus*, and *G. cuvieri* was also swept into that latter genus [22, 28, 43]. Finally, the fossil teeth classically assigned to *Galeorhinus* and *Physodon* were considered as female and male morphs of *Physogaleus* spp. [24, 26]. This suspicion was raised because the type specimen of *Physodon muelleri* was re-identified as a male of *Scoliodon laticaudus* [44].

Our observations contradict the referral of *G. cuvieri* to *Physogaleus* and the implied sexual dimorphism in dentition in this species. *G. cuvieri* is best referred to the living genus *Galeorhinus* on the basis of a diagnostic combination of tooth and body characters. The living *Galeorhinus* does not exhibit distinguishable sexual dimorphism in tooth forms [30, 38, 45], and neither does *G. cuvieri*. This is borne out by the fact that a male (MGGC 1976) and a female (MCSNV T 1124) of *G. cuvieri* could be identified using the same diagnostic combination of tooth and body characters. However, the status of *G. cuvieri* does not determine whether or not other tooth-based species of *Galeorhinus* represent the carcharhinid *Physogaleus* [28] or whether or not the teeth of *Physogaleus* represent discrete sexual morphotypes [24, 26].

The assignment of *G. cuvieri* to the living genus *Galeorhinus* reflects the observation that this taxon is morphologically closer to *G. galeus* than to any other living triakids. A suite of characters in *G. cuvieri* — both in the tooth morphology and body proportions — follows that of the genus *Galeorhinus* (listed in **Diagnosis**) [25] and differs from the type *G. galeus* only in those diagnostic to the species (listed in **Diagnosis**; Figs. 4, 5 and 9). These characters diagnostic to *G. cuvieri* also differentially distinguish the taxon against other species of *Galeorhinus* (outlined in **Dentition**). The congeneric status with *G. galeus* implies the existence of the genus for at least 50 million years. If *G. glickmani* from the Cenomanian of Russia also represents a congeneric species [28, 31], the chronological range may even extend well into the Late Cretaceous times. With mosaic distribution of tooth characters among the living and fossil species of *Galeorhinus*, it would be difficult to distinguish *G. cuvieri* at the generic level to the exclusion of all other species of *Galeorhinus*. Optimally sorting the observed morphological variations at the generic level would require a comprehensive systematic reassessment of the fossil *Galeorhinus* spp. At this point, the conservative approach is to reflect apparent morphological proximity with *G. galeus* in the generic assignment of *G. cuvieri*.

### The bolca as a possible nursery habitat for *G. cuvieri*

The extant *G. galeus* inhabits warm temperate and tropical waters on continental shelves [25]. Juveniles tend to occupy inshore ‘nursery’ habitats such as protected bays or estuaries, whereas adults occur across the continental shelf. [30, 38, 45–50] *G. cuvieri* may have had similar ecology to *G. galeus*. All six specimens of *G. cuvieri* with whole body preservation from Bolca likely represent sexually and somatically immature juveniles (discussed in **Age and Size Estimates**; Table 1). MGGC 1976 (length: 92 cm) is the largest, and five other coeval specimens range from 54 cm (BM B70) to 92 cm (MCSNV T 1124) in total length (Figs. 2 and 8; Additional file 1: Table S1). If all of the specimens of *G. cuvieri* are indeed sexually immature, the Pesciara di Bolca locality may have been the nursery habitat.

Consistent with the nursery interpretation, the Pesciara di Bolca assemblage deposited in a shallow lagoonal setting bordered by active fluvial and coastal systems and by coral reefs [8, 9, 51]. These environmental features agree well with the modern nursery areas (generally protected, nearshore/inshore habitats) for various coastal selachians, including triakids [52, 53]. In such nearshore/inshore habitats, juvenile sharks tend to form a diffuse community of mesopredators feeding on similarly sized preys [54]. A nursery area may be identified on the basis of three criteria: (1) relative occurrence of the species in the habitat/area versus outside; (2) duration of occupying the habitat/area; and (3) repeated use of the habitat/area across years [52]. None of these criteria can be evaluated quantitatively for *G. cuvieri* with the current sample. Although the occurrence of likely juvenile individuals alone does not constitute direct support for the Bolca locality as a nursery for *G. cuvieri*, circumstantial evidence is consistent with that interpretation.

### Trophic interactions in the Eocene coral reefs

The stomach content of MGGC 1976 indicates that *G. cuvieri* fed on *S. bolcensis*. The extant *Galeorhinus* frequently preys upon *Sphyraena* as a significant component of its diet that heavily relies on reef fish communities [25, 30, 38, 45, 46]. *Sphyraena* itself is a secondary to tertiary consumer in low-latitude coral reefs [47, 55]. Therefore, the stomach content provides a rare glimpse of feeding relationships near top of the trophic network in this coral reef fish community near the exit of the Early Eocene Climatic Optimum [2, 15].

The *Galeorhinus*-*Sphyraena* feeding interaction at higher trophic levels underscores modern features of the fish community of Bolca. The high functional diversity of herbivorous Bolca fishes is linked to post–Cretaceous increase in morphological disparity of acanthomorphs, and these are considered as important factors in shaping the Cenozoic coral reef communities [3, 20]. Disparity analyses suggest that the colonization followed a classic niche–filling scenario [3, 18–21, 56]. The invasion of herbivorous acanthomorphs into the coral reef habitats must have been accompanied by correlated shifts at higher trophic levels, and the *Galeorhinus*-*Sphyraena* association supports that prediction.

Coupled with the ecomorphological comparison of primary consumers [18–20, 56], the *Galeorhinus*-*Sphyraena* association provides evidence for deep conservation of the food web structure in modern coral reef communities. The late Ypresian age for the Bolca locality suggests that this particular feeding interaction occurred under the shift of marine vertebrate fauna across the Paleocene–Eocene thermal maxima. The challenges to testing this interpretation are: (a) poor age constraints for both the predator and prey lineages; and (b) the paucity of pre-Ypresian marine Lagerstätten to link the modern features of the Bolca vertebrate fauna unambiguously to the climatic pattern. As for the *Galeorhinus*-*Sphyraena* association, the trophic relationship cannot be presumed earlier than the earliest occurrence of either predator or prey lineage. The uncertain identification notwithstanding, teeth referable to the Triakidae occur in the Cenomanian of Ukraine and, possibly, the Hauterivian of England [31, 57]. It is difficult to constrain the origin of *Galeorhinus* or that of the Triakidae further. This is partly because the tooth morphology is highly heterogeneous among triakids [24, 28] and partly because molecular evidence is equivocal about whether or not *Galeorhinus* is nested within the Triakidae [58–60]. As for *Sphyraena*, *S. bolcensis* provides the hard minimum calibration point for the split between sphyraenids and their sister groups [61]. So the trophic association is as deep into fossil record as the Sphyraenidae. This is consistent with the hypothetical link to the Climatic Optimum, but the causal relationship is non-falsifiable with the data available at hand.

At the faunal level, the paucity of pre-Ypresian Lagerstätten poses a challenge to associate modern features of the Bolca fauna with the exit of the Climatic Optimum [3, 20]. Elsewhere in the world, the stomach content of the scombrid teleost *Auxides huberti* from the contemporaneous late Ypresian locality of Senegal indicates that this tuna–like extinct scombrid fed on another extinct scombrid, the mackerel-like *Eoscomber senegalicus* [62]. Although this association has no congeneric parallel in modern marine ecosystem as in between *Galeorhinus* and *Sphyraena*, it reinforces the occurrence of modern feeding relationships at higher trophic levels near the exit of the Climatic Optimum. However, this interpretation has its basis in the marine Lagerstätten from the post–Climatic Optimum; little evidence exists in the immediately preceding stages to support or reject it. As such, the link to global climatic patterns awaits a test through increased sampling in the Paleocene-Eocene assemblages.

## Additional file

Additional file 1: Table S1. A table of measurements for selected specimens of *Galeorhinus cuvieri*. Numbers for metric traits refer to those in Fig. 8. Measurements were made in cm. Specimen housed in the Museo Geologico Giovanni Capellini, Bologna, Italy: MGGC 1976a, b (slab and counter slab): a juvenile male (Figs. 2, 3, 4, 5, 6, and 7). Specimen housed in the Muséum national d'Histoire naturelle, Paris, France: MNHN F Bol516 (holotype specimen of *G. cuvieri*) (Fig. 8A). Specimen housed in the Museo di Geologia e Paleontologia, Padova, Italy: MGP-PD 8871C, 8872C (slab and counter slab): a juvenile (Fig. 8B). Specimens housed in the Museo Civico di Storia Naturale in Verona, Italy: MCSNV VII B96, B97 (slab and counter slab): a juvenile (Fig. 8C); MCSNV T.1124: a juvenile female (Fig. 8D-F). **Table S2.** Body lengths (in cm) of triakid taxa that are known to reach sexual maturity in the size range represented by specimens of *Galeorhinus cuvieri*. The data are based on ref. [1]. (DOCX 95 kb)
