# Supplementary material for: An exceptionally preserved Eocene shark and the rise of modern predator–prey interactions in the coral reef food web
Source: Zoological Lett. 2016 Apr 1;2:9. doi: 10.1186/s40851-016-0045-4 (PMC4818435; doi:10.1186/s40851-016-0045-4)
Supplement: Additional file 1: Table S1. — A table of measurements for selected specimens of Galeorhinus cuvieri. Numbers for metric traits refer to those in Fig. 8. Measurements were made in cm. Specimen housed in the Museo Geologico Giovanni Capellini, Bologna, Italy: MGGC 1976a, b (slab and counter slab): a juvenile male (Figs. 2, 3, 4, 5, 6, and 7). Specimen housed in the Muséum national d'Histoire naturelle, Paris, France: MNHN F Bol516 (holotype specimen of G. cuvieri) (Fig. 8A). Specimen housed in the Museo di Geologia e Paleontologia, Padova, Italy: MGP-PD 8871C, 8872C (slab and counter slab): a juvenile (Fig. 8B). Specimens housed in the Museo Civico di Storia Naturale in Verona, Italy: MCSNV VII B96, B97 (slab and counter slab): a juvenile (Fig. 8C); MCSNV T.1124: a juvenile female (Fig. 8D-F). Table S2. Body lengths (in cm) of triakid taxa that are known to reach sexual maturity in the size range represented by specimens of Galeorhinus cuvieri. The data are based on ref. [1]. (DOCX 95 kb) [file 40851_2016_45_MOESM1_ESM.docx]

**Table S1** A table of measurements for selected specimens of *Galeorhinus cuvieri*. Numbers for metric traits refer to those in Fig. 8. Measurements were made in cm. Specimen housed in the Museo Geologico Giovanni Capellini, Bologna, Italy: MGGC 1976a, b (slab and counter slab): a juvenile male (Figs 1-4). Specimen housed in the Muséum national d'Histoire naturelle, Paris, France: MNHN F Bol516 (holotype specimen of *G*. *cuvieri*) (Fig. 5A). Specimen housed in the Museo di Geologia e Paleontologia, Padova, Italy: MGP-PD 8871C, 8872C (slab and counter slab): a juvenile (Fig. 5B). Specimens housed in the Museo Civico di Storia Naturale in Verona, Italy: MCSNV VII B96, B97 (slab and counter slab): a juvenile (Fig. 5C); MCSNV T.1124: a juvenile female (Fig. 5D-F).

| Metric traits  (Fig. S1) | MGGC 1976 | MNHN F Bol516 | MCSNV T.1124 | MGP-PD 8871C | MCSNV VII B96 |
| --- | --- | --- | --- | --- | --- |
| 1 | 92 | 70 | 92 | 69 | 79 |
| 2 | 17 | 15 | 16 | 13 | 15 |
| 3 | 46 | ? | 46 | 34 | 44 |
| 4 | 29 | ? | 30 | 22 | 20 |
| 5 | 21 | 16 | 22 | 15 | 22 |
| 6 | 50 | ? | 52 | 40 | 43 |
| 7 | 40 | 42 | 41 | ? | 32 |
| 8 | 51 | ? | 51 | 39 | 37 |
| 9 | 4 | ? | 4 | 3.5 | ? |
| 10 | 15 | ? | 13 | 9 | ? |

**Table S2.** Body lengths (in cm) of triakid taxa that are known to reach sexual maturity in the size range represented by specimens of *Galeorhinus cuvieri*. The data are based on ref. [1].

| **Taxon** | **Male** | **Female** |
| --- | --- | --- |
| *Mustelus fasciatus* | >62 | ? |
| *Mustelus antarcticus* | >68 | 80 |
| *Mustelus asterias* | 78-85 | 85 |
| *Mustelus californicus* | 57-65 | 70 |
| *Mustelus canis* | 82 | 90 |
| *Mustelus dorsalis* | 43 | 43 |
| *Mustelus griseus* | 62-71 | 80 |
| *Mustelus henlei* | 52-66 | 51-63 |
| *Mustelus higmani* | 43 | 48 |
| *Mustelus lenticulatus* | 78-89 | 79-113 |
| *Mustelus lunulatus* | 70-83 | ? |
| *Mustelus manazo* | 62-70 | 62-70 |
| *Mustelus mento* | 65-76 | 86-90 |
| *Mustelus mosis* | 63-67 | ? |
| *Mustelus mustelus* | 70-74 | 80 |
| *Mustelus norrisi* | 58 | 65 |
| *Mustelus punctulatus* | 50-55 | 60 |
| *Mustelus schmitti* | 48 | 55-60 |
| *Triakis scylium* | 99-108 | ? |
| *Triakis megalopterus* | 140-142 | 140-150 |
| *Triakis semifasciata* | 70-119 | 110-129 |
| *Hemitriakis japanica* | 85 | 81-102 |

**Reference for Table S1**

1. Compagno LJV: *Sharks of the Order Carcharhiniformes*. Caldwell, N.J: The Blackburn Press; 2003.
